# How does the British public understand mental health? A qualitative analysis of open-text responses

**DOI:** 10.1177/00207640211052174

**Published:** 2021-10-25

**Authors:** Megan Arnot, Miranda Wolpert, Ethan Greenwood

**Affiliations:** 1 Department of Anthropology, University College London, UK; 2 Division of Psychology and Language Sciences, University College London, UK; 3 Wellcome Trust, London, UK

**Keywords:** Mental health, understanding, lay, qualitative

## Abstract

**Background:**

An individual’s understanding of mental health can influence their attitudes towards those experiencing mental health problems, and also impact their response to any mental health problems they experience. However, what the lay public understand about mental health is not well explored in existing research.

**Aims:**

This study aims to gain a deeper insight into what the general public understand by the term ‘mental health problem’.

**Methods:**

Data were taken from a large-scale representative sample of adults from Great Britain (*n* = 2,708). A thematic analysis was carried out on an open-text question which asked people what they understood by the term ‘mental health problem’.

**Results:**

Six themes were identified in the thematic analysis, which included understanding mental health through thinking about cause and effect, thinking about the location of mental health problems in the body, the universality and variation of mental health problems, reflections on lived experience and identifying a specific mental health problem.

**Conclusion:**

The analysis suggests that there are many diverse ways the public conceptualises mental health. The themes identified may be useful for future quantitative analyses, and also may suggest how information about mental health can be best communicated to the public.

## Background to the study

In recent years, an increasing number of people have reported experiencing a mental health problem of some kind (Häfner, 1985; Kessler et al., 2005; Klerman & Weissman, 1989; Ngui et al., 2010; Richter et al., 2019). What has caused this increase is highly complex, with many mental illnesses likely being the result of multiple factors – such as genetics, socio-political factors and childhood trauma (Ni et al., 2020; Torjesen, 2019) – interacting with one another through various mechanisms such as epigenetics (Kumsta, 2019), gene-environment interactions (Assary et al., 2018) and familial intergenerational inheritance (Boursnell, 2011). It may also be that the increase in mental health problems is in part due to the increased reporting of symptoms that may have previously been taboo, and therefore concealed (Jorm et al., 2017). Regardless of the cause, globally, slightly more than 1 in 10 people have lived with a mental health condition (Richie & Roser, 2018), and there is evidence that this number is rising (Richter et al., 2019), with research showing that this has been accelerated by coronavirus disease-2019 pandemic (Nochaiwong et al., 2021). As a result, mental health problems are increasingly recognised as an important public health concern.

Findings from existing research suggest that how people understand their own and others’ mental health conditions affects their behaviour, in terms of how they may respond to others with a mental health condition, and also how they may seek and respond to treatment if they were experiencing a problem themselves (Dang et al., 2020; Goldney et al., 2005; Jorm, 2000; Jorm et al., 1997). For example, people who conceptualised depression as being a personal weakness rather than a mental health problem were more likely to try and deal with the problem alone (Jorm et al., 2006); while those who attributed depression to ‘biological’ factors such as genetics were observed to be positively disposed to taking anti-depressants (Carter et al., 2017). In addition to this, different people’s understanding of mental health has been found to impact their response to treatment, with those who understand mental health problems to derive from more biological factors (e.g. genetics) adhering to medical treatment (e.g. anti-depressants) for a longer period of time; while those who perceive mental health problems to be caused by social factors appear to prefer therapy over a more medicalised treatment, like drugs (Carter et al., 2017).

As one’s understanding of mental health impacts identification, help-seeking behaviours and treatment, it is critical to gain knowledge about what the general public do understand about mental health problems. Though there has been a focus on gauging the levels of mental health literacy amongst the public in recent years, much of this research has focussed on a specific facet, such as what people think the best treatments are, and what individuals classify as symptoms (Butlin et al., 2019; Choudhry et al., 2016). Further, much of this research has been carried out using quantitative methods, which can somewhat be restrictive when trying to gauge public understanding of a topic; or using vignette-based approaches, which have been criticised based on inconsistencies in how they are used within research and a lack of ecological validity (Sai & Furnham, 2013).

To build on previous research, in this study, we aim to gain a more general understanding of how the public understands the term ‘mental health problem’ through conducting an exploratory qualitative analysis on a sample of open-text responses to a single question. Through doing so, we hope this will identify how mental health is conceptualised by non-specialists, and also identify avenues for future academic research.

## Materials and methods

### Participants

Data used in this study were taken from the fourth wave of the Wellcome GB Monitor (National Centre for Social Research, 2018), with respondents being drawn from the National Centre for Social Research (NatCen) online panel. Members of the NatCen panel are recruited from the British Social Attitudes (BSA) survey, which randomly selected participants aged 18 or over from across Britain to partake in a face-to-face interview. Panellists who joined the BSA in 2017 and 2018 were invited to take part in the Wellcome GB Monitor, with fieldwork taking place in November and December of 2018. A total of 4,775 invitations were sent out, and 2,708 interviews achieved (57% response rate). For full findings and methodology see https://wellcome.ac.uk/reports/wellcome-monitor-2018.

### Analysis

As part of the Wellcome GB Monitor, participants were asked a number of questions to gauge what they think the aims of mental health research are, what researchers within the field do, and finally what they understand by the term ‘mental health problem’. In this study, we conduct an exploratory analysis of people’s open-text responses to the following question:

*‘Mental health problems can be thought about in many different ways. We are interested in finding out what you understand by the term ‘mental health problem’. In your own words, what do you understand by the term ‘mental health problem’?’*

To answer this question, respondents were able to offer as much or as little information as they wanted. An open-text question is beneficial for data collection here because, in the likely absence of any specialist or specific knowledge, a set of multiple-choice answers might force responses that were not necessarily an accurate reflection of what the individual actually understood. In other words, when asked what they understand by ‘mental health problems’, if pre-set responses were provided, people might have considered all options as plausible and ticked them all. Thus, asking an open-text response enables any analyses to glean authentic and instinctive responses from the public.

An exploratory thematic analysis was carried out on the participant responses to the above question, which is a qualitative approach to examining research data, and used to identify themes within the data. Procedural guidelines provided by Braun and Clarke (2006) for conducting a thematic analysis were followed, in which one first familiarises themselves with the data, then generates preliminary codes and themes, then reviews the themes and finally defines and names the themes. Line-by-line coding of the responses was carried out using NVivo (Version 12 for Mac) (QSR International Pty Ltd, 2020), which involves coding the open-text responses based on the identified theme. As recommended by Elo and Kyngäs (2008), this was an iterative process, with new themes being included in the coding framework as they emerged. As the thematic analysis was being carried out, the coding of different participant responses was compared to ensure coding consistency.

## Results

### Participant characteristics

**Figure 1.**
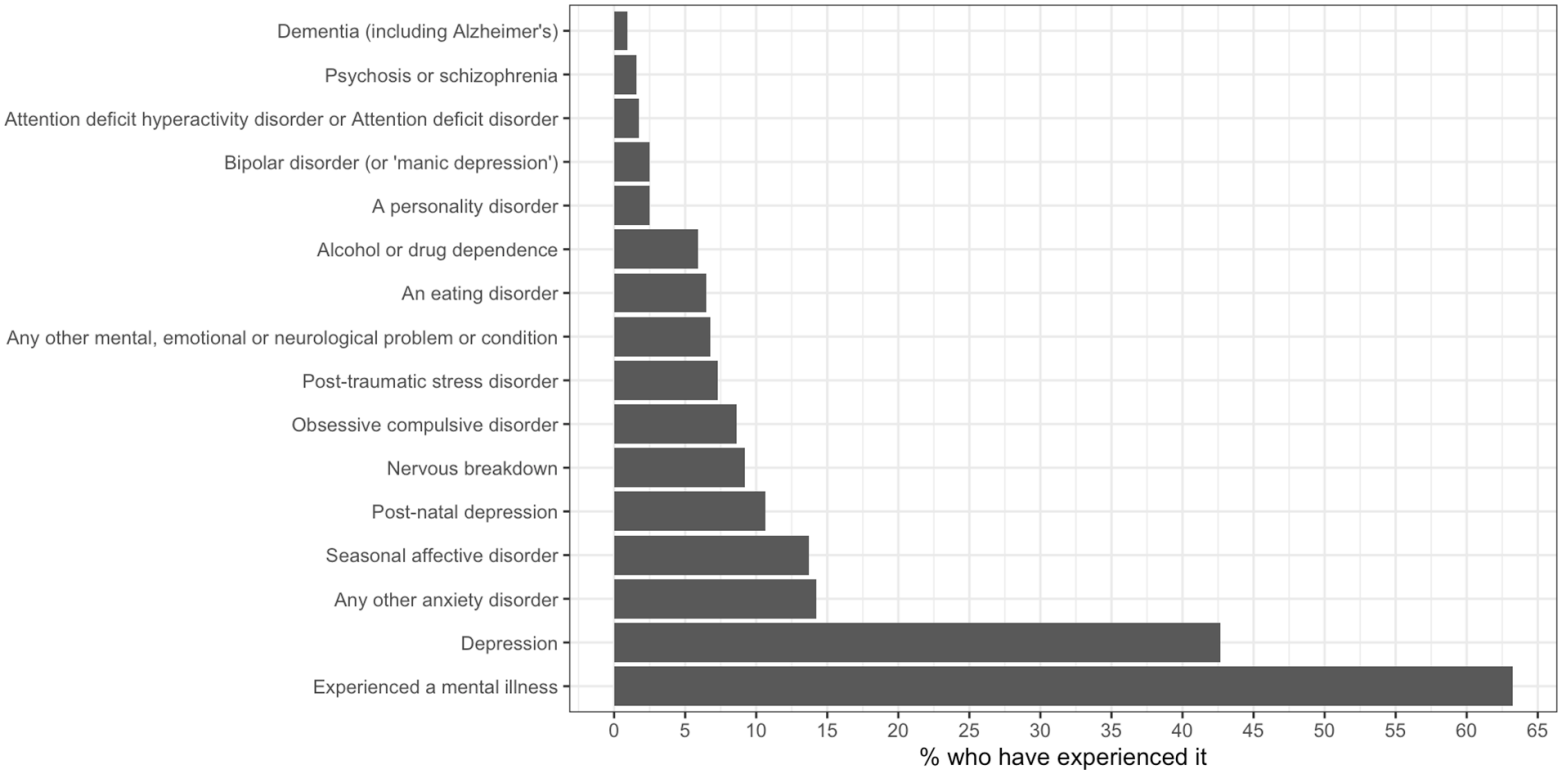
Percentage of respondents who have experienced various mental health problems (*n* = 2,708).

Amongst the 2,708 interviewees, there was a slight bias towards females within this sample (58%), while those aged below 29 were under-represented compared to the other age groups (9%). The majority of respondents were educated to a degree level or above (43%), were married or living as married (62%) and had an equivalised household income of more than £2,000 a month (35%). The sample was extremely ethnically biased, with 86% of people identifying as being white (either white British, or another white background). The majority of respondents had experienced a mental health problem of some kind (63%), with depression being the most common, followed by anxiety and seasonal affective disorder (see Figure 1). However, it should be noted that the variable pertaining to the participants’ experience of mental health problems is self-reported, and so may or may not relate to a formal diagnosis given by a professional. Full descriptive statistics are shown in Table 1.

**Table 1.** Characteristics of participants who responded to the open-text question (*n* = 2,708).

	*n* (%)
Sex	
Male	1,145 (42.3)
Female	1,563 (57.7)
Age	
18–29	237 (8.8)
30–39	447 (16.6)
40–49	459 (17.1)
50–59	508 (18.9)
60–69	536 (19.9)
70+	504 (18.7)
Education	
Degree or equivalent, and above	1,167 (43.3)
A levels or vocational level 3 or equivalent, and above	567 (21.0)
Other qualifications below A levels or vocational level 3 or equivalent	462 (17.1)
Other	200 (7.4)
No qualification	298 (11.1)
Marital status	
Married/civil partnership/living with partner	1,649 (61.3)
With a partner you do not live with	140 (5.2)
Separated/divorced	307 (11.4)
Widowed/surviving partner from a same-sex civil partnership	173 (6.4)
Single (never married/never in a civil partnership)	421 (15.7)
Ethnicity	
White British (English/Welsh/Scottish/Northern Irish)	2,163 (86.0)
Any other White background	145 (5.8)
Mixed or multiple ethnic groups	31 (1.2)
Asian or Asian British	84 (3.3)
Black or Black British	52 (2.1)
Other	41 (1.6)
Equivalised income	
<£800	534 (20.3)
£800–£1,250	548 (20.8)
£1,250–£2,000	637 (24.2)
>£2,000	913 (34.7)
Experienced a mental illness	
No/prefer not to say	996 (36.8)
Yes	1,712 (63.2)

### Themes identified

In total, six themes were identified in the responses, which are defined and illustrated in Table 2. These included participants demonstrating knowledge through listing or identifying specific mental health problems (Theme 1), stating that mental health problems lie on a spectrum (Theme 2), reflecting on lived experience (Theme 3), thinking about the cause and effect of mental health problems (Theme 4), considering the location of mental health problems (Theme 5), and addressing the idea that mental health problems are universal and could happen to anyone (Theme 6). These themes are not mutually exclusive, and responses often included multiple themes in different combinations. In addition to these themes, ~6% of respondents stated that they did not know what a mental health problem was, or gave a vague or irrelevant answer.

**Table 2.** Summary of identified themes in response to the question ‘Mental health problems can be thought about in many different ways. We are interested in finding out what you understand by the term “mental health problem.” In your own words, what do you understand by the term “mental health problem”?’, examples of such and key findings from the thematic analysis.

Theme	Example	Summary of findings from theme
1. Mentioning specific conditions	‘Low mood, anxiety, depression, stress’	Understanding of mental health problems demonstrated through just listing conditions without any more reflection
	‘Issues in the mind which prevent normal functioning in day to day life or thoughts of harming oneself or others, including fear, phobias, anxiety and depression as well as psychotic conditions’	
2. Mental health problems lying on a spectrum	‘Anything from mild to severe conditions, such as anxiety, depression, PTSD, OCD, schizophrenia, etc.’	Depression and anxiety considered to be less serious than schizophrenia
	‘Any psychological issues, ranging from general anxiety to more serious diagnoses like bipolar’	The ‘less serious’ illnesses are more manageable/treatable than those that are ‘more serious’
3. Reflecting on lived experience	‘I have a personal understanding of mental health problems as I suffer from [mental health conditions]. Which I attend counselling regularly for. . .’	Own experience associated with self-reporting a good understanding of mental health problems
		Concern about impact implicit in answers
4. Cause and effect	‘Chemical brain imbalance, for ex lower serotonin leading to anxiety or depression’	Cause often dichotomised into a biological versus social cause
	‘something that is affecting your everyday ability to function, can be stress, anxiety, etc’	Impact generally perceived to be negative, and a deviation from what is considered normal
5. Mental health problems being in the mind or brain	‘People who has an unstable mind’	Perceived division between the body and mind/brain
	‘Brain related illness, thought processing, stress, anxiety, depression’	Mental health problems still considered differently to other illnesses
6. Universal in prevalence	‘It is a very severe illness which is causing problems to 99% of the population these days, in things like stress or anxiety. . .’	Mental health problems are becoming more common because of today’s society and because of awareness
	‘Everybody at some point in life will suffer from a mental health problem. . .’	No one is impervious to them

#### Theme 1: Mentioning specific condition

Across all the responses, one of the most common themes was for people to name specific conditions within their response, with approximately one-fifth of respondents doing so. The naming of these conditions was sometimes weaved into the discussion, possibly to demonstrate a specific understanding of what a mental health problem is.

*‘A medical problem which does not have a physical cause, such as mood disorders like anxiety and depression, which causes the individual distress’.*

Additionally, many people’s entire open-text response just included a list of mental health conditions, without substantiating their understanding with further information.

*‘Depression/anxiety/OCD/loneliness/suicidal thoughts/bullying/sch/schizophrenia/dementia’*

*‘Post Traumatic Stress Disorder (PTSD), depression, anxiety, agoraphobia and other phobias, hyper arousal’.*

Of those who did mention a specific illness, depression was most common, followed by anxiety.

#### Theme 2: Mental health problems lying on a spectrum

Implicit within the previous theme, but also a category in its own right, was the tendency to state that mental health lies on a spectrum and naming specific conditions was often used as a means of illustrating this spectrum. This spectrum can be dichotomised, with some people differentiating between long-term and short-term illnesses, and others stating that mental health problems range in terms of severity. Frequently, depression and anxiety were classified as being less serious illnesses that might be experienced by everyone at some point in their lives, while schizophrenia and psychosis were often named as being ‘more serious’ disorders that were more long-term, and also rarer amongst the general population.

*‘Depression, anxiety, bipolar disorder, anorexia and other eating disorders. more serious mental disorders/psychiatric issues like schizophrenia’*

*‘Mental health problems concern anybody suffering from some form of issue which affects their ability to function as an fully fledged member of society. Such issues can range from minor, such as lower forms of depression, but can also entail things such as schizophrenia which mark much more serious deficiencies. Such things are can be treatable and are preventable at certain intervals but a problem implies it is not been met at such a level yet’.*

#### Theme 3: Reflecting on lived experience

When asked how they understood the term ‘mental health problems’, many respondents chose to reflect on their own lived experience to demonstrate understanding. This might have been through themselves experiencing a mental health problem, through watching close friends and family experience one, or occasionally discussing where they have seen mental health problems in the general population. When they did have first-hand experience of mental health problems (e.g. through personally having a mental health problem or seeing a close friend or family member experience one), they generally reported that they understood mental health problems very well.

*‘I understand it quite well having suffered from [mental health condition] most of my life also family members have [mental health condition] I know and understand people that suffer bi polar, adhd, cod’.*

*‘I have a personal understanding of mental health problems as I suffer from [mental health conditions]. Which I attend counselling regularly for. . .’*

*‘I am a counsellor so I understand mental health very well. I have suffered myself with emotional and psychological distress and I help people on a weekly basis with their struggles. I believe that the DSM should be abolished along with labelling people. I also believe that mental health education should be part of the curriculum in every school’.*

Implicit within the responses that did reflect on experience was the consideration of the impact of the mental health not only on themselves (e.g. losing confidence, changing their behaviour and moods) but also those around them (e.g. making them worry, harming them).

*‘Its very difficult, I’ve [had a mental health problem] for the [a number of] years and it has made me feel very ill. I’ve lost my confidence and feel very lonely. I have not responded to how many different drugs I’ve been put on, but they have made me feel like a zombie. My GP’s have been marvellous, they may have thought I had something more serious as I had lost so much weight. I’ve had tests and all they have shown up is my stress and anxiety’.*

*‘Mental health is something I suffer from since a young age it runs in my family but was also cause by my up bringing. [I experience many symtoms]. I don’t feel normal and my children are picking up traits they are learning from me and it hurts. I excersised for years to keep myself from hitting rock bottom but now I need tablets x’*

#### Theme 4: Cause and effect

Many people’s responses reflected on what causes mental health problems, and also the impact they can have both on the individual and those around them. Not everyone who considered cause also spoke about impact, and vice versa.

##### Theme 4

1: Cause.

Of those who demonstrated their understanding of the term mental health problems through discussing their aetiology, there was a slight dichotomisation: some people attributed mental health problems to more biological processes, while others stated they were the product of more social phenomena. Where the causal process mentioned was more biological in nature, people typically mentioned genetics and brain chemistry.

*‘Any genetic, developmental or behavioural issue causing problems for the person reporting symptoms’*

*‘Something to do with the brain, chemicals in the brain not working properly’*

*‘Unable to function normally due to thoughts in someone’s head. . ..probably as a result of some traumatic event’*

Amongst these individuals, there was the narrative that mental health problems are predetermined and permanent, therefore largely outside of the actors control and something over which they have little responsibility.

*‘a disability of different types . Something you are born with and have to live with for rest of your life’*

In contrast, those that attributed a more social cause to mental health problems suggested that they were somewhat avoidable. Here, respondents typically stated that mental health conditions were caused by a social process, such as a major traumatic event or childhood abuse. This therefore suggests that in the absence of this stressor the individual would have otherwise been mentally well.

*‘Someone who may have had a challenging upbring that has effected them. Or anyone who has suffered life changing events. Or suffered anxiety or stress as a result of a massive change or shock to their usual lifes pattern. It can be something big or small. . .. it depends on just how resilient the person is to cope and equally as important, the network of support they have around them. If no support its easy to understand how the negativity can spiral’.*

One common theme amongst those attributing a socially derived cause was the stress of ‘modern life’ or ‘everyday life’.

*‘Individuals that cannot cope with everyday life due to many modern day factors’.*

*‘Cause of much mental health problems is modern day living’.*

This suggests people think that our current environment is not ideal for optimal mental health and, that while social factors are modifiable, the way that life currently is means that mental health problems are – to some extent – an inevitability.

Very few people clearly stated that they understood mental health problems to be the result of an interaction between biological and social factors. Even amongst respondents who included both within their response, there was still a dichotomisation between the two, with the suggestion that while some mental health problems are caused by social or environmental circumstances, other are purely the result of biological factors. Typically, depression and anxiety (which had previously been linked in Theme 2 to being ‘less serious’) were recognised as being the product of social factors; while illnesses like schizophrenia (identified as ‘more serious’ in Theme 2) were attributed to being the product of a more biological, or predetermined, factors, like genetics.

*‘When you cannot cope with normal daily life or certain aspects of it.also when a physical problem within the brain may cause issues that a person is unable to control or deal with’*

*‘Some mental health problems are due to a chemical imbalance in our brain. Some mental health problems are because of past experiences or trauma or how we feel about ourselves’*

*‘issues arising that have a detrimental affect to a persons well being. stress and anxiety being most prevalent in a fast paced society. Eating disorders created by image obsession. Genetic or medical abnormalities such as schizophrenia etc’*

##### Theme 4

2: Effect.

Many participants demonstrated their understanding of the term ‘mental health problems’ by reflecting on their outcomes, and commenting on their symptoms, their impact on the sufferer’s wellbeing and personality, and also their effect on those close to the actor. These impacts were generally described in a negative light, by suggesting that those with mental health problems may not be able to experience day-to-day life in the same way as those who are mentally well.

*‘Mental health problems include anxiety, depression, psychological well-being, addictions, individuals not able to function in their day to day activities and having a negative effect on there well being’.*

Running through these responses was also the idea that mental health problems result in people behaving in a way that deviates from what is considered normal. This suggests that, despite their prevalence in society, there may still be a stigma against the outward symptoms of mental illnesses.

*‘Thoughts or actions that repeatedly impact on a person rendering them unable to conduct their life and function in a ‘normal’ way in society’.*

#### Theme 5: Mental health problems being in the mind or brain

Numerous of the responses highlighted that mental health problems are not comparable to other illnesses (deemed as being ‘physical’ illnesses) such as cancer.

*‘Chronic and debilitating health issues that are not apparently ‘physical’ and manifest in depression, anxiety, phobias, psychological disorders that are debilitating and diminish quality of life’.*

Rather, they were considered to be an illness of the mind or the brain.

*‘Mental Health Problems are Invisible diseases of the brain. They can cause problems just without outward symptoms’.*

*‘Any disorders of the mind which prevent you from living your normal everyday life’*

This implies that the public are somewhat separating mind and body, with mental health problems not being understood by the public in the same was as ‘illnesses of the body’.

#### Theme 6: Universality

Finally, people also demonstrated that they understood mental health problems to be common, with many people experiencing them.

*‘a mental health problem is a situation where a person does not feel right, whether that is, being depressed or wanting to die. These are very common and can be very mild to very severe but everyones mental health problems are different to each others’.*

They were also often perceived to be indiscriminate in nature, with people stating that anyone could experience a mental health problem, regardless of factors like social status and general health, meaning that no one is immune from experiencing one.

*‘There are many forms of MH problems that people may suffer from bad experiences through having a bad childhood, trauma, bereavement etc. Stressful work conditions, worrying about family, health. All can lead to episodes of mental health problems. Which could happen to anyone from any parts of society’.*

*‘A mental health problem is just as important as a physical health problem. It can happen to anyone at anytime. It includes the sub categories anxiety and depression that seemingly more and more people are being diagnosed with. I think this is due to our new understanding as a society of what it means’.*

## Discussion

### An overview of the themes

The findings reported above provide an insight into how the British public’s understanding of mental health problems is constructed. A primary finding was the tendency of people to name or list specific illnesses, with many choosing to name illnesses as a means of illustrating their understanding (Theme 1). Amongst these respondents, depression was most commonly named, followed by anxiety. Interestingly, this is in line with the respondents’ experience of mental health problems (Figure 1), and the distribution of mental health problems in the United Kingdom, with depression being the most frequently reported mental illness (Bridges, 2015), and also the disorder with the most research funding (Woelbert et al., 2019). It is also notable that stress was identified as being a mental health problem at a high frequency despite not being a psychiatric diagnosis itself. While stress itself is not a mental health problem, it is closely related to them through both causing them, worsening existing ones (Bovier et al., 2004; Caspi et al., 2003; Shankar & Park, 2016), and it is also known to aggravate other health problems (Ahmad & Zakaria, 2015; Arnot et al., 2021; Leserman et al., 2000; Steptoe & Kivimäki, 2012). The tendency to classify stress as a mental health condition has been observed elsewhere, where doing so was associated with more negative attitudes towards mental health (Rüsch et al., 2012). This is likely illustrative of an overlap in peoples’ minds between general mental wellbeing and mental health problems (Payton, 2009). Further, the conceptualisation of ‘stress’ as a mental health problem may somewhat explain the high levels of participants reporting having ever experienced mental health problems (see Limitations for more discussion on this), as, if this everyday experience is seen as a mental health problem, then it is expected that most people would have experienced it.

Implicit within Theme 1 (but also a theme in its own right) was the idea that mental health problems lie on a spectrum of seriousness and severity (Theme 2), with depression and anxiety often being named as mental health problems that are more common and less severe, while other disorders, such as schizophrenia and bipolar, were considered to be more serious. This demonstrates an understanding that the term ‘mental health problem’ is an umbrella term that encompasses a number of conditions or states, many of which differ greatly in nature.

The idea that specific mental health problems differ from one another in many ways was also addressed by respondents who spontaneously shared ideas on what causes mental health problems in their open-text responses (Theme 4.1). Based on the sample of people included in this study, it appears that the lay understanding of what causes mental health problems can somewhat be divided into categories (i.e. biological, psychosocial). This means of categorisation appears be in line with two general models of lay beliefs about mental health problems that have been proposed (Furnham & Bower, 1992). The first model is the ‘medical model’, where mental disorders are considered similar to other illnesses, with any outwards symptoms having a biological pathway (Rabkin, 1974). The second model is the ‘psychosocial model’, in which it is suggested that mental health problems are caused by psychological and environmental/social factors (Sarbin & Mancuso, 1972). These models are useful for classifying lay beliefs about mental health, and in many ways mirror divisions in beliefs amongst mental health experts (Butlin et al., 2019). They may also have wider implications in regards to attitudes towards those with mental health problems and also treatment preferences. In terms of treatment, those experiencing depression who think the illness is caused by more biological factors (such as genetics) have been observed to take anti-depressants for a longer amount of time when compared to patients who attribute depression to social factors (Carter et al., 2017). Similarly, if people think that their social environment is causing their mental health problems, then they are more likely to favour a social form of intervention – such as therapy – as treatment as opposed to a biomedical one (Alahmed et al., 2018). Beliefs related to what causes mental illness not only impact how patients access and respond to treatment, but also how they are treated by health professionals in the first place. Clinicians have been observed to deem medication as being a more effective route of treatment for mental illnesses they thought were primarily predetermined and the product of genetic factors, such as schizophrenia and bipolar; whereas psychotherapy was the preferred treatment for illnesses such as bulimia and anxiety, to which social causes were more often attributed (Ahn et al., 2009). Medical professionals have also been observed to vary in empathy towards patients depending on their own preconceived notions of what causes the illness. Here, evidence has been found suggesting that less empathy is shown towards patients with illnesses they thought were biologically, rather than socially, caused (Lebowitz & Ahn, 2014).

As well as the cause of the illness, people also considered the effect mental health problems have on the individual experiencing them, and also the impact it has on those around them (Theme 4.2). The general consensus was that the effects of mental illnesses are unfavourable: many stated that they prevent people from being able to live their day-to-day life in a ‘normal’ way, and that friends and family of someone with a mental illness also experience a detrimental impact. This negative perception was prevalent throughout people’s responses, despite many people claiming that mental illnesses are common today, with no one being impervious to them and that they are somewhat an inevitability of the stresses of everyday life (Theme 6). This suggests that, despite mental health problems being seen as a common phenomenon, their outward symptoms are still viewed as abnormal and possibly still have a stigma attached to them as a result. Many were aware of the negative perception of having a mental illness, with one respondent stating: ‘*. . . they are very common and still have some stigma attached to them*’.

People also reflected on their own experience to demonstrate their understanding of mental illness (Theme 3). As these people had first-hand experience of mental health problems, they generally did not offer a definition of the term ‘mental health problems’ but rather offered a deeper analysis based on lived experience. It was often reported that their experience of mental illness was hard due to the public stigma, the impact of the illness on those close to them and also the concealed nature of the illness: ‘. . . *because it is not associated with a bandage or a visible condition or remedy it can be extremely hard for friends and families to appreciate just how hard the impact can be*’. The non-physical nature of mental health problems was also addressed in Theme 5, in which we identified that people commonly communicated their understanding of mental health problems by describing them as being an illness of the mind or brain, that’s separate from other illnesses, with mental health problems often being contrasted with cancer or a broken leg. The mind-body dualism is rooted in work by Descartes (1996), who argued that the mind and body are separated entities that could each exist by itself. Despite the advances in understanding how ‘the mind’ works, and what causes mental health problems, it has been noted that there’s still a tendency for people to view the mind as a separate entity from the body (Demertzi et al., 2009; Miresco & Kirmayer, 2006), and that when mental health problems are seen as separate from other ‘bodily illnesses’, there is a greater stigma attached to them (Raese, 2015).

### Implications

This research highlights the variety of ways in which the public understand and conceptualises mental health. As stated previously, one’s understanding of mental health can affect their ability to recognise a condition, their treatment decisions and their attitude towards others with mental health problems. Hence, knowing not only what people understand about mental health, but *how* they understand it, will be of importance for public health initiatives. By communicating information to the public via themes that they already understand – for example, by focussing on the fact mental health lies on a spectrum – it may improve how it is received. Further, the findings presented in this paper may also highlight areas that require better communication to the public, such as the cause of mental health problems. For example, as stated very few people stated that they understood that mental health conditions have various causes that are generally an interaction of both social and biological factors, rather, most stated that it was either/or. Results from this study may also be of use to quantitative researchers aiming to conduct more specific research into mental health literacy. Themes identified here may provide a framework from which to derive more narrowly focussed questions for a deeper understanding of how the public currently understand mental health.

### Limitations

It should be noted that this sample is likely not completely representative of the British population. Based on demographics from 2018, younger people under the age of 30 were under-represented in this sample (Office for National Statistics, 2018a), while those who were highly educated (Office for National Statistics, 2018b) were over-represented. The former has been linked with poorer mental health literacy (Farrer et al., 2008), while the latter is associated with a better general understanding of mental health, and also an improved ability to communicate understanding of mental health, which would be of use in the context of open-text questions.

Approximately three in five people in this sample reported having ever experienced a mental health problem, which is a greater proportion that the general British public (NHS, 2021), meaning there is likely a sample bias. It has been shown that those with personal experience of mental health problems have a better understanding (Dahlberg et al., 2008), and hence it is likely that the understanding and responses presented within this sample are not an accurate representation of how the average Briton understands mental health. As previous stated, the high proportion of people within this sample with experience of a mental health problem may be due to the tendency to class stress as a mental health problem; but it may also be because question pertaining to own experience of mental health problems states ‘Do you think that you have personally experienced any of the following’, meaning that it likely includes people who have self-diagnosed as experiencing a mental health problem, in addition to those who had a formal diagnosis, thus increasing the sample size. Research on mental health literacy has shown a tendency, particularly amongst the younger generation, to ‘over-identify’ depression (Farrer et al., 2008), and the prevalence of those reporting depression within this sample could reflect this; however, the participants were primarily aged 30 and over so this may not be the case.

Another limitation is in the nature of the responses. Even though there was no restriction on the length of the participant’s responses, on the whole they were brief and limited to just one or two sentences. As a result, it is unlikely we were able to capture the full range and nuances in people’s understanding of mental health. Further, because the question was open-text, it also meant that there were some differences in interpretation of the question (as highlighted in the variety of themes we were able to isolate), and so while the responses provide us with an idea of the range of ways in which people understand mental health, further investigation into each specific theme would be required to gauge the specifics of peoples encapsulation of mental health.

## Conclusion

We have shown that there are many ways the public understand mental health and that this understanding can be communicated in multiple ways, such as through diagnosis, cause and impact. While this analysis was exploratory in nature, it can be used to inform policymakers and those involved in science communication when constructing health promotions concerning mental illness to effectively communicate ideas to the public. Of course, the responses analysed here cannot be expected to completely capture the people’s understanding of mental health as it’s a highly complex subject matter. The open nature of the question means that it could have been answered in a multitude of ways, and therefore just because someone demonstrated their answer through solely discussing cause (for example), it does not mean that that is the limit of their understanding. Future research could aim to use the themes identified in this research as guidance for a more specific understanding of how the public understands mental health.
